# Effect of long-term feeding of the Obudu natural honey and table sugar-sweetened diets on obesity and pro-inflammatory biomarkers in rats

**DOI:** 10.1186/s40795-019-0327-2

**Published:** 2020-02-03

**Authors:** Item Justin Atangwho, Chidimma Emmanuel Ibeneme, Godwin Eneji Egbung, Emmanuel Ibeneme, Margaret Akpan Eno, Promise Nwankpa

**Affiliations:** 1 Department of Biochemistry, College of Medical Sciences, University of Calabar, P.M.B., 1115 Calabar, Nigeria; 2 Department of Medical Laboratory Science, College of Medical Sciences, University of Calabar, P. M. B., 1115 Calabar, Nigeria; 3 Department of Medical Biochemistry, Imo State University, Owerri, Nigeria

**Keywords:** Obudu cattle ranch honey, Table sugar, Adipokines, Insulin, Tissue necrosis factor – α (TNF-α)

## Abstract

**Background:**

This study investigated long-term effect of the Obudu honey on selected biomarkers of energy storage regulation, compared to table sugar.

**Methods:**

Fifty Wistar rats assigned to 5 groups of 10 rats each, were fed rat chow only (NC), 8% table sugar (S8%), 16% table sugar (S16%), 10% honey (H10%) and 20% honey (H20%) diets respectively, for 29 weeks. On dry weight basis, the percentages of table sugar and honey for each level of incorporation were equivalent. Diet intake, body weights and fasting blood glucose (FBG) were measured fortnightly. At the end of the study, serum glucose, insulin, leptin and tissue necrosis factor – α (TNF-α), wet weight of white adipose tissues (WAT) were measured.

**Results:**

After an initial adjustment to the diets, there was no significant difference in diet consumed by female and male subgroups, except the female group fed H20% which was consistently lower than the NC and the corresponding S16% fed group (*P* < 0.05). Both honey and sugar incorporated diets caused significant body weight gain in the female animals compared to NC; an effect which was higher with the honey than sugar, and depended on the level of each sweetener used as well as feeding duration (*P* < 0.05). Furthermore, S8% and S16% diets increased leptin concentration in the female rats, by 35.8 and 45.3% respectively compared with NC and by 63.8 and 40.5% compared to H10% and H20% respectively (*P* < 0.05). Also, the S8% and S16% diets significantly increased serum insulin in the female subgroups compared to the corresponding honey-sweetened diets; and in both male and female rats when compared to NC (*P* < 0.05). Lastly, the S8% and S16% diets also caused a dose-dependent increase of TNF-α in both female and male rats compared to the H10% and H20% diets and the control (*P* < 0.05).

**Conclusion:**

Data obtained from the study associated table sugar with obesigenic and inflammatory mechanisms more than the Obudu honey, particularly in the females. However, the data did not exempt the honey from obesigenic effect. The effects were subtle and may require a longer time to precipitate obesity.

## Background

Overweight and obesity have reached epidemic proportions and are major causes or risk factors of several chronic diseases including Type 2 diabetes, cardiovascular diseases, obstructive sleep apnoea, non-alcoholic fatty liver, malignancies and various complications [1]. Overweight and obesity which were once considered problems of the developed countries, are now on the increase in the developing countries, especially in the urban areas [2] leading to the coexistence of over-nutrition with under-nutrition in the developing world [3]. This rising epidemic in the developing countries is as a result of profound changes in society, behavioural pattern and nutrition transition especially in urban areas [4]. One of such life style changes that contributes majorly to the looming obesity epidemic is the change in eating habit - increased consumption of energy dense foods, (with high levels of calorie-rich sweeteners) [5].

Sweeteners including honey and table sugar have always been used to enhance the taste of food and palatability [6]. These sweeteners comprised mainly simple carbohydrates (sucrose, glucose, fructose, etc), hence significantly contribute “extra” calorie to the diet. Simple carbohydrates also have high glycaemic index which does not only affect blood glucose, insulin and lipid response but also affects the appetite, energy intake and body weight [7]. Consequently, these “extra” calories can lead to positive energy balance and ultimately obesity as consumption of more than the needed calories is one of the major causes of overweight and obesity [8]. On the strength of this, the extra calorie addition by sweeteners and its implications on weight related pathologies have generated serious concerns amongst health scientists and consumers on the choice and preference of sweeteners, particularly the most commonly used, honey and table sugar.

Given the difference in chemical compositions of honey and table sugar, honey is usually considered a healthier food sweetener than table sugar. However, this notion has been challenged by research reports which show that the non-sugar nutrients are only in very minute amounts [9]. The implication is that for any desirable nutritional effect, the honey must be in substantial amounts, which would in turn unduly increase the calorie intake of the individual, hence a high predisposition to overweight and subsequently, obesity if this positive energy balance state is prolonged [10].

Furthermore, in the recent past, there were reports suggesting the hypoglycaemic effect of honey, which led to the recommendation of honey as a better substitute to table sugar for diabetics [11]. Contrariwise, a recent scientific report forewarns Type 2 diabetics against the use of honey in preference to table sugar, because of its propensity to raise triacylglycerol (body fat) levels [12]. Additionally, a study in Iran showed that 8-week honey consumption in diabetics increased glycated haemoglobin in diabetic subjects, indicating severity of complications [13]. Previously too, a strong positive correlation between obesity and excess calorie intake via consumption of sugar-sweetened foods and beverages including aerated soft/energy drinks and other synthetic fruit juices were shown [14]. However, in the Australian paradox report, Australian sugar consumption was found to decrease over the same period that obesity increased [15].

These emerging facts and findings have heightened a medical debate as to which of the two sweeteners is healthier and safer, given the widespread daily use of these sweeteners and the alarming rate of the global obesity epidemic. According to Erejuwa [16], a long-term study on the effect of honey consumption may help to ascertain the validity of the aforementioned claims and counterclaims or provide some clarifications to the contradictions.

Consequently, this study compared the effect of the Obudu natural honey sweetened diet with table sugar diet on biochemical biomarkers of energy storage regulation in male and female Wistar rats. To our knowledge, no such evaluation had been carried out previously with the Obudu Cattle Ranch natural honey, which is widely distributed in the South-South Region and other parts of Nigeria.

## Methods

### Materials

Natural honey was obtained from the Obudu Cattle Ranch in Obanliku Local Government Area, Cross River State; and white granulated sugar was purchased from Golden Penny Company (Apapa, Lagos State), Nigeria. Accu-check Active glucometer with its corresponding test strips were obtained from Mannheim, Germany. Rat Insulin and tissue necrosis factor-alpha (TNF-α) ELISA analytical kits were obtained from EastBioPharm, Hangzhou, China, whereas rat leptin ELISA kits were obtained from BioVendor, Czech Republic.

### Animals

Fifty (50) Wistar rats; 25 males and 25 females, weighing 120 to 140 g obtained from the animal house of the Department of Genetics and Biotechnology, University of Calabar, Calabar, were used for the study. The rats were housed in wooden cages with wire mesh lid, in the animal facility of the Department of Biochemistry, University of Calabar, where the research was carried out. They were allowed two weeks of acclimatization, under room temperature (27 ± 2 °C) and 12-h light/dark cycles before the commencement of the experimental feeding procedures. Prior to commencement of the experiments, fasting blood glucose (FBG) and body weight were measured and used as the bases of assigning animals into five (5) groups of ten rats each (5 males and 5 females). The male and female rats belonging to the same group were kept in separate cages throughout the experimental period (i.e. five rats per cage) to avoid mating. The average body weight per group at the outset of the experiments was 138 ± 4 g, *n* = 10. The animals were allowed access to drinking water and the diets ad libitum. The use of animals and research procedures were approved by the Faculty Animal Research Ethics Committee, Faculty of Basic Medical Sciences (FAREC-FBMS), University of Calabar (Approval Number: 014B20117).

### Diet formulation and feeding of the experimental rats

The table sugar and honey were incorporated in the rats’ diets in proportions that simulate average sugar composition of common sweetened foods and beverage [17]. The table sugar and honey were incorporated by wet mixing with the rat chow (w/w) and re-pelleting. The diets included control diets, diets containing 8 and 16% sugar, 10 and 20% honey as shown in Table 1 below. The percentages of sweeteners were equivalent based on dry weight i.e. 8 and 16% table sugar being equivalent of 10 and 20% honey, respectively, based on dry weight; given that honey is reported to contain 20% water [18]. The diets were prepared every other day to ensure freshness and avoid spoilage, and fed to the rats according to the schedule in Table 1. Furthermore, leftover and spilled diets were carefully collected and weighed in order to determine the rats’ diet intake i.e. the difference between initial diet supplied and the leftover. The feeding of the experimental rats with diets lasted for 29 weeks. Within this period, the rats’ body weights (g) and FBG were measured fortnightly using a digital electronic balance and a glucometer (Accu-check Active, Mannheim, Germany) respectively.

**Table 1:** Experimental design and feed formulation

S/No	GROUP	NUMBER OF RATS	FEED COMPOSITION
1	Normal control	10	Rat chow (100%)
2	Sugar-fed І	10	Sugar (8%) + Rat chow (92%)
3	Sugar-fed ІІ	10	Sugar (16%) + Rat chow (84%)
4	Honey-fed І	10	Honey (10%) + Rat chow (90%)
5	Honey-fed ІІ	10	Honey (20%) + Rat chow (80%)

### Termination of animal feeding and collection of samples

At the end of the 29-week feeding, the rats were made to fast overnight (8 pm to 6 am), but allowed free access to drinking water. The final in-life measurements including body weights and FBG were carried out. The percentage change in body weights (%) was calculated thus:

[Final body weight - Initial body weight × 100] / [Initial body weight]

The rats were anaesthetized with chloroform vapour inhalation in a closed chamber. These anaesthetized rats were dissected (laparatomy) and with sterilized syringes whole blood was collected via cardiac puncture into non-heparinized sample tubes. The blood was allowed to clot for about two hours after which it was centrifuged at 3000 rpm for 10 min, and serum collected. The serum was stored frozen until used for biochemical analyses. Afterwards the rat carcasses were dissected and liver, heart, kidneys and white adipose tissues (peri-renal and epididymal) were surgically removed, blotted with filter paper and their wet weight measured. The relative weight was calculated thus: w/W × 100%. Where w = wet weight of an organ and W = final body weight of the rat before sacrifice.

### Serum biochemical analyses

The serum obesity markers, insulin, leptin and pro-inflammatory biomarker (TNF-α) were determined using EastBioPharm and BioVendor rat assay kits by Sandwich-ELISA method. The assay procedures used were according to the manufacturer’s instructions. In brief, the method uses a double-antibody sandwich enzyme-linked immunosorbent assay (ELISA) to determine the levels of rat insulin, leptin and tumour necrosis factor-α in samples.

### Statistical analysis

The data were expressed as the mean ± SEM (*n* = 5). The data were analysed by one-way ANOVA followed by a Least Square Difference (LSD) post hoc test for evaluation of significance between mean values of treatment groups and the controls. The SPSS software version 20.0 (IBM SPSS Inc., Chicago, IL, USA) was used. Differences at *P* < 0.05 were considered significant.

## Results

### Diet consumption

**Fig. 1:**
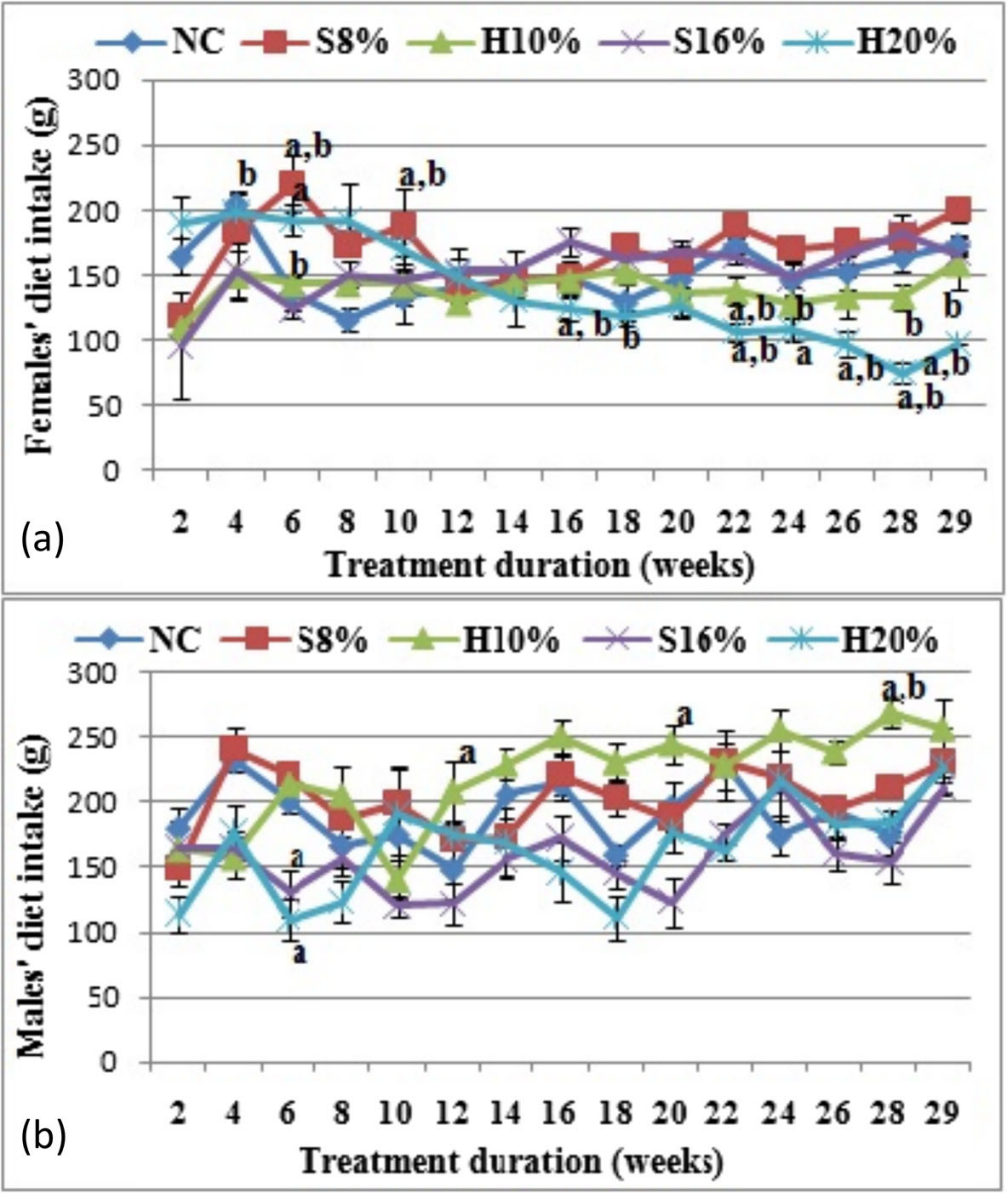
Diet consumption (g) of female (**a**) and male (**b**) rat groups fed natural honey and table sugar sweetened diets respectively. NC = normal control, S8% = 8% sugar sweetened diet group, S16% = 16% sugar sweetened diet group, H10% = 10% honey sweetened diet group and H20% = 20% honey sweetened diet group. a = *P* < 0.05 vs. NC and b = *P* < 0.05 vs. corresponding energy groups. Values represent the means ± SEM, *n* = 3–5

The diet consumed by the female and male rat groups measured over the 29-week administration is shown in Fig. 1 (a) and (b) respectively. The results showed no significant effect of the sweetened diets on consumption pattern, except the female rats fed H20% diet which increasingly ate less compared to the normal control (NC) and the corresponding S16% fed group (*P* < 0.05) till the end of the study (Fig. 1 a).

### Body weights changes

**Fig. 2:**
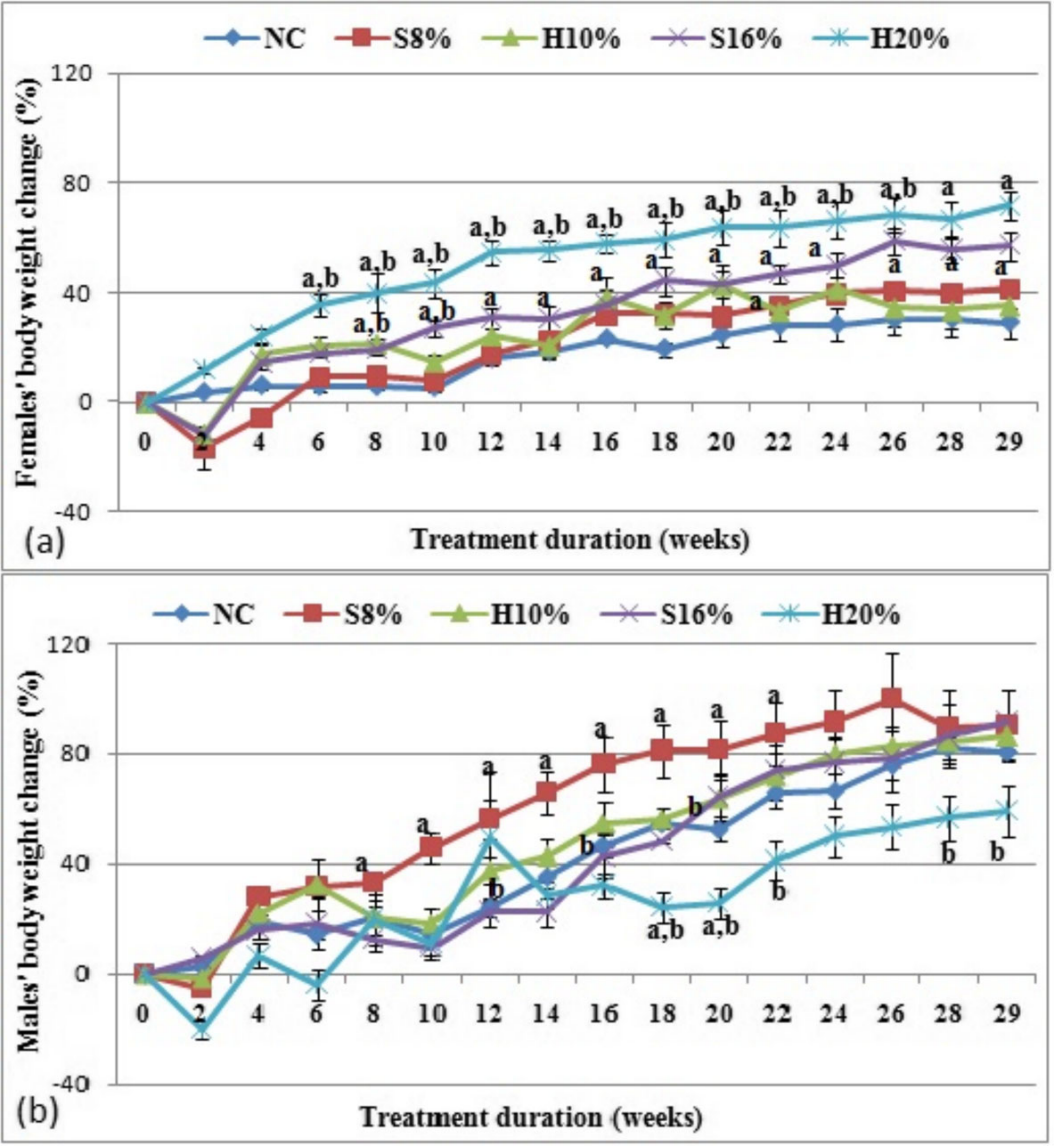
Percentage changes in body weights of female (**a**) and male (**b**) rat groups fed natural honey and table sugar sweetened diets respectively. NC = normal control, S8% = 8% sugar sweetened diet group, S16% = 16% sugar sweetened diet group, H10% = 10% honey sweetened diet group and H20% = 20% honey sweetened diet group. a = *P* < 0.05 vs. NC and b = *P* < 0.05 vs. corresponding energy groups. Values represent the means ± SEM, *n* = 3–5

The percentage changes in body weights of female and male rat groups measured over the 29-week study duration are shown in Fig. 2 (a) and (b) respectively. Both honey at 20% (H20%) and table sugar at (S16%) caused higher body weight gain continuously in the female rat groups than that in the control (*P* < 0.05); an effect found to be higher with the honey fed (H20%) rats than the sugar fed (S16%) and depended on the % of sweetener used and duration of feeding (*P* < 0.05). In the male counterpart body weight of H20% fed rats was seen to decrease from week 18 till the end of the study.

### Fasting blood glucose variations

**Fig. 3:**
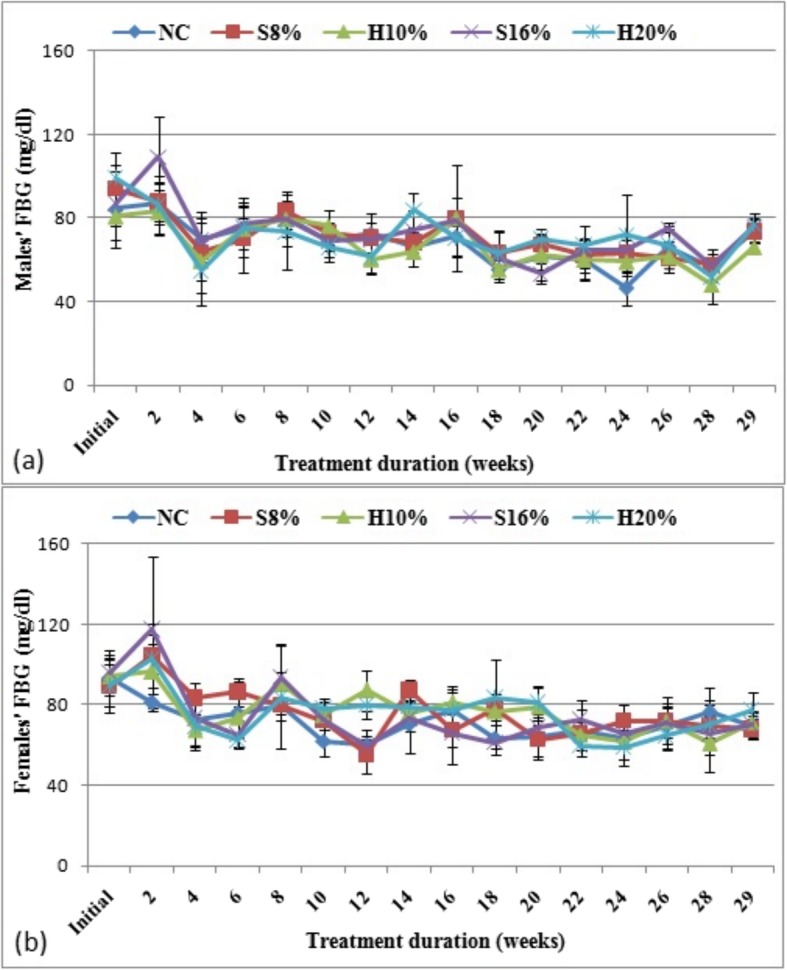
Fasting blood glucose, FBG, (mg/dl) concentration in female (**a**) and male (**b**) rat groups fed with natural honey and table sugar sweetened diets respectively. NC = normal control, S8% = 8% sugar sweetened diet group, S16% = 16% sugar sweetened diet group, H10% = 10% honey sweetened diet group and H20% = 20% honey sweetened diet group. a = *P* < 0.05 vs. NC and b = *P* < 0.05 vs. corresponding energy groups. Values represent the means ± SD, *n* = 3–5

Fasting blood glucose (FBG) concentrations of female and male rat groups measured over the 29-week study period are shown in Fig. 3 (a) and (b) respectively. Aside occasional transient changes, no significant effect of the sweetened diets on the FBG were observed in both male and female sub-groups.

### Relative organ/tissue weights

**Fig. 4:**
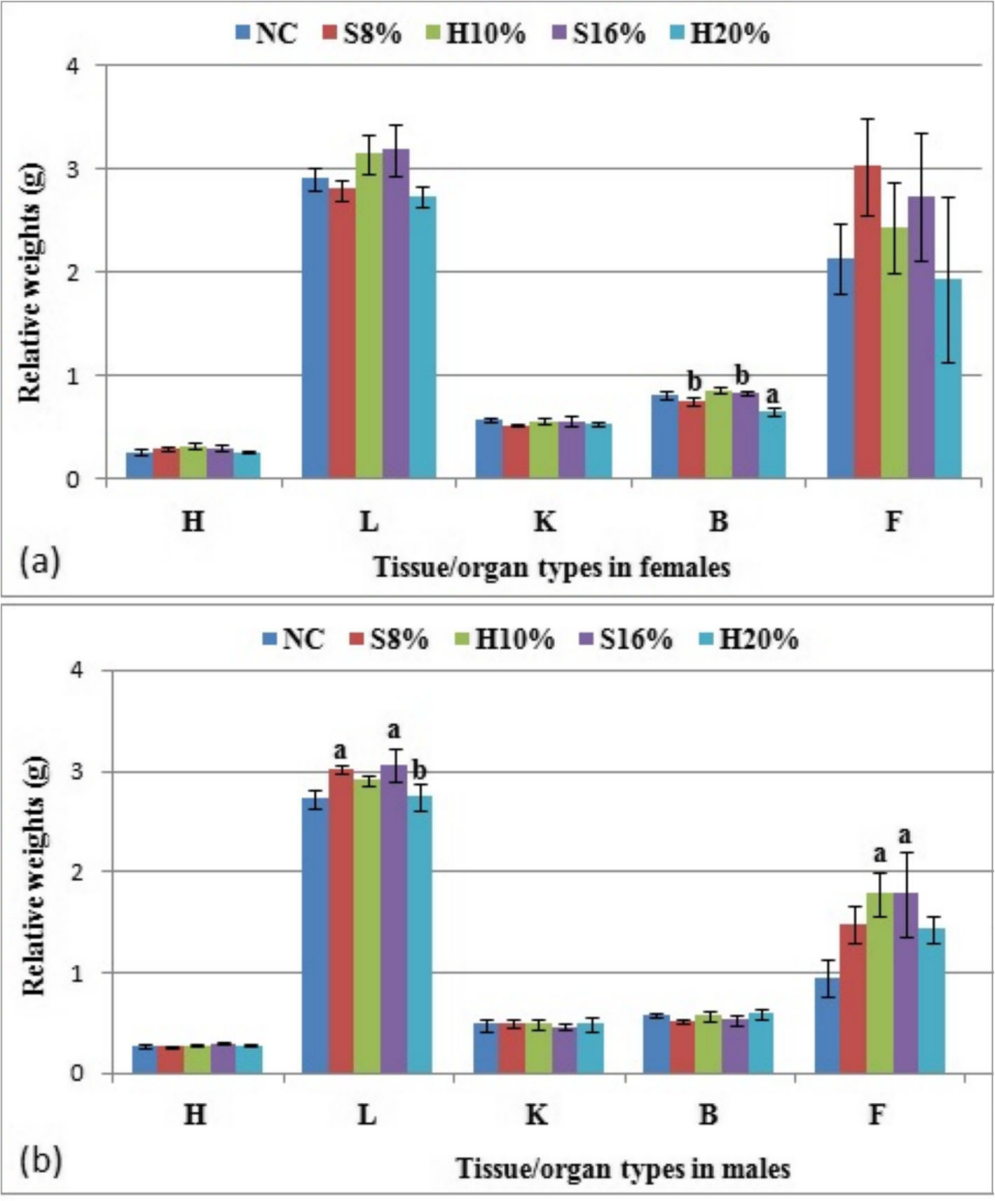
Relative tissue/organ weights (g) of female (**a**) and male (**b**) rat groups fed natural honey and table sugar sweetened diets respectively. H = Heart, L = Liver, K = Kidneys, B = Brain and F = Fat. NC = normal control, S8% = 8% sugar sweetened diet group, S16% = 16% sugar sweetened diet group, H10% = 10% honey sweetened diet group and H20% = 20% honey sweetened diet group. a = *P* < 0.05 vs. NC and b = *P* < 0.05 vs. corresponding energy groups. Values represent the means ± SEM, n = 3–5

The relative wet weights of selected internal organs of the female and male rats fed the study diets for 29 weeks are shown in Fig. 4 (a) and (b) respectively. From the results, there were no significant differences in the relative weights of measured organs - hearts, livers, kidneys and brain in the female rats fed table sugar sweetened diets when compared to the corresponding honey fed groups or the normal control (*P* > 0.05). The white adipose tissue weights of test groups fed 8 and 16% sugar sweetened diets were found to increase by 19.6 and 29.3% compared to the 10 and 20% honey sweetened diets respectively. In the male counterparts, the relative liver weights of sugar-fed groups i.e. S8% and S16% both increased significantly in a dose-dependent manner, by 9.7 and 11% compared to NC (*P* < 0.05). However, when compared to the corresponding honey sweetened diets, there was significant increase in the 16% sugar fed group only, relative to H20% (*P* < 0.05). Also, significant increase in the weight of WAT in groups H10% (46.7%) and S16% (46.9%) compared to NC were recorded (*P* < 0.05). Other measured tissue weights in the male rats namely, brain, kidneys and heart showed no significant impact of the sweeteners (*P* > 0.05).

### Serum insulin concentration

**Fig. 5:**
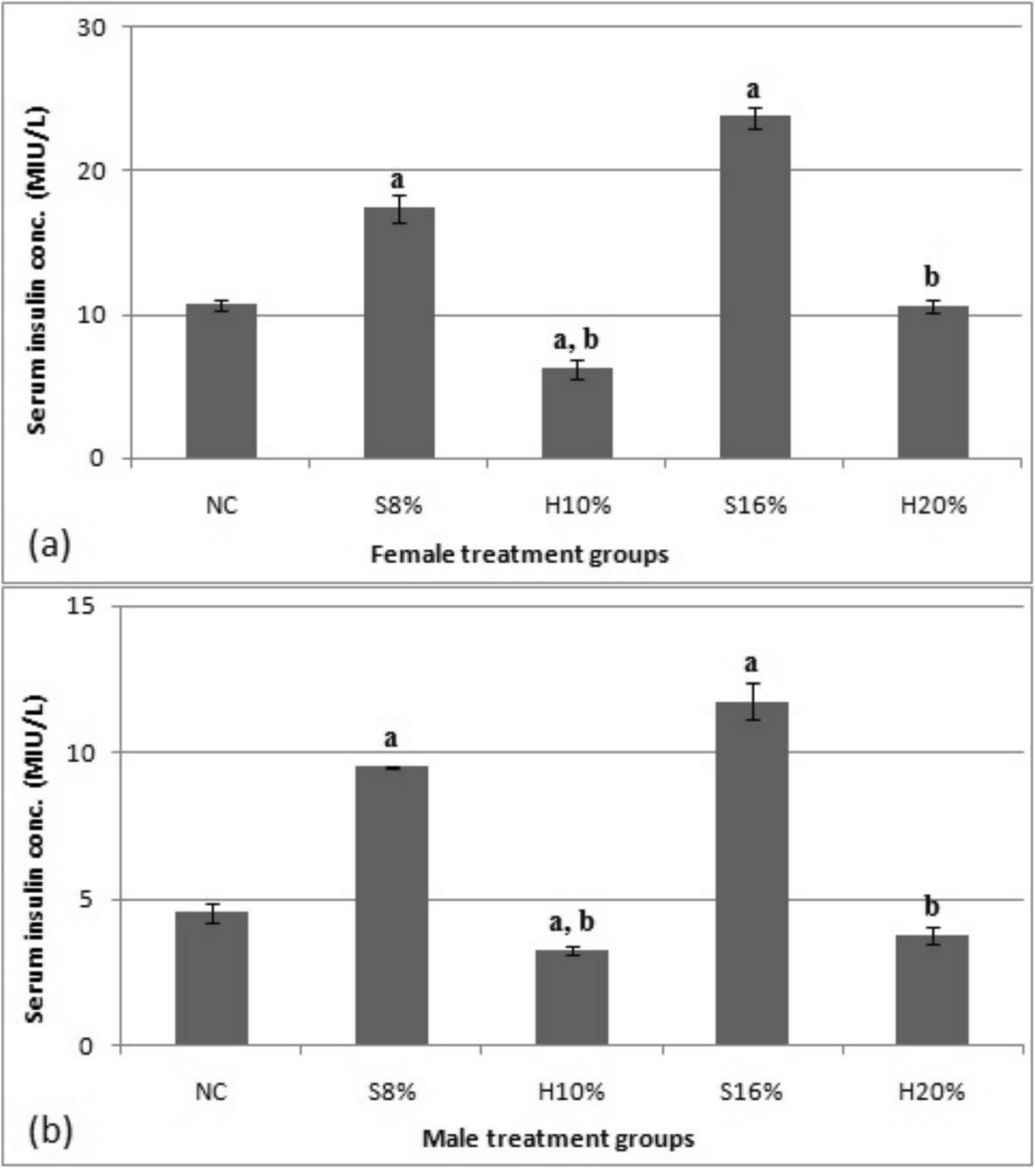
Serum insulin concentration (MIU/L) of female (**a**) and male (**b**) rat groups fed natural honey and table sugar sweetened diets. NC = normal control, S8% = 8% sugar sweetened diet group, S16% = 16% sugar sweetened diet group, H10% = 10% honey sweetened diet group and H20% = 20% honey sweetened diet group. a = *P* < 0.05 vs. NC and b = *P* < 0.05 vs. corresponding energy groups. Values represent the means ± SEM, n = 3–5

Data showing the effect of 29-week feeding of natural honey and table sugar sweetened diets on serum insulin concentration in female and male study subjects is depicted in Figs. 5. The results obtained for the female rats showed that serum insulin concentration (MIU/L) was increased in the sugar-fed groups viz. S8% and S16% by 64.2 and 55.3% respectively compared to their corresponding honey-fed groups (viz. H10% and H20%); and by 39.0 and 55.1% respectively compared to normal control (*P* < 0.05). These profound effects of sugar and honey sweetened diets were entirely similar in the male counterparts.

### Serum leptin concentration

**Fig. 6:**
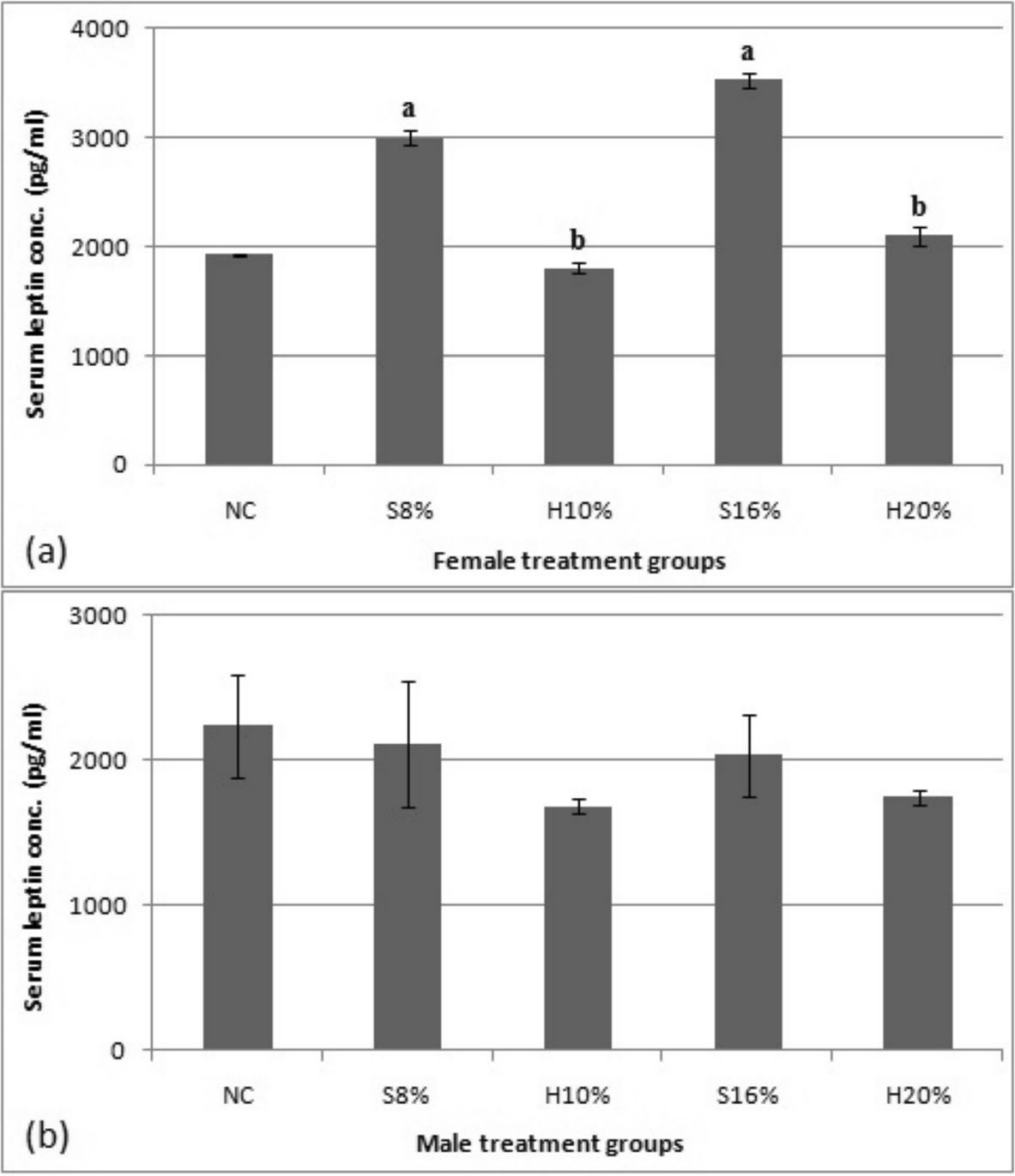
Serum leptin concentration (pg/ml) of female (**a**) and male (**b**) rat groups fed natural honey and table sugar sweetened diets. NC = normal control, S8% = 8% sugar sweetened diet group, S16% = 16% sugar sweetened diet group, H10% = 10% honey sweetened diet group and H20% = 20% honey sweetened diet group. a = *P* < 0.05 vs. NC and b = *P* < 0.05 vs. corresponding energy groups. Values represent the means ± SEM, n = 3–6

The results of the effect of 29-week experimental feeding of sugar and honey sweetened diets on serum leptin concentration in female and male rats are shown in Fig. 6. Twenty-nine (29) week feeding of sugar-sweetened diets, S8% and S16% was found to increase leptin concentration (pg/ml) in the female rats by 35.8 and 45.3% respectively compared with the normal control (*P* < 0.05). Moreover, compared to the honey-fed groups (H10% and H20%) the 8 and 16% sugar sweetened diets caused 63.8 and 40.5% increases in serum leptin concentration respectively (*P* < 0.05). However, in the male rats, neither the sugar nor the honey sweetened diets impacted significantly on the leptin concentration (*P* < 0.05).

### Serum tumour necrosis factor alpha (TNF-α) concentration

**Fig. 7:**
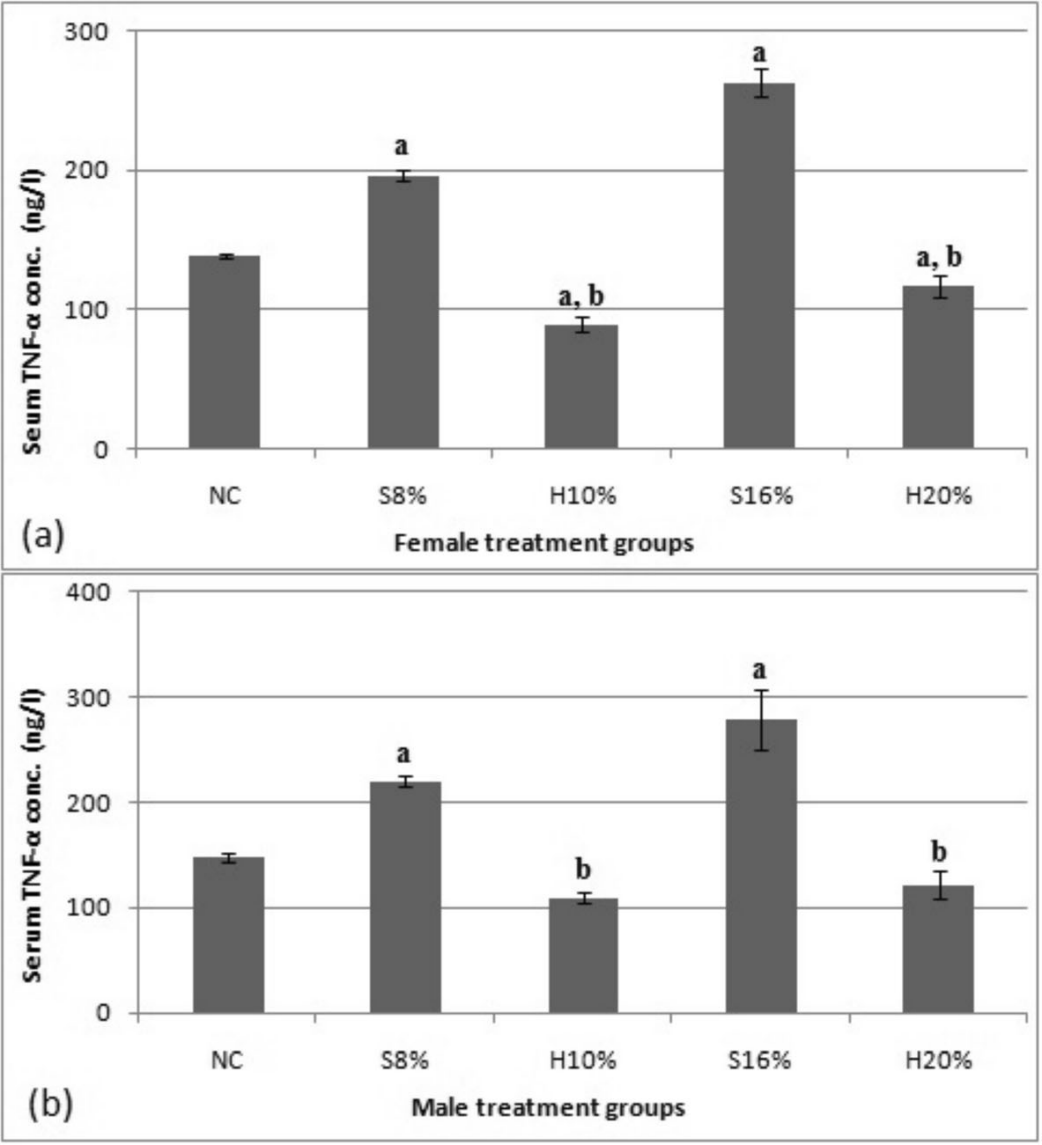
Serum tumour necrosis factor alpha (TNF-α) concentration (ng/L) in female (**a**) and male (**b**) rat groups fed with natural honey and table sugar sweetened diets.NC = normal control, S8% = 8% sugar sweetened diet group, S16% = 16% sugar sweetened diet group, H10% = 10% honey sweetened diet group and H20% = 20% honey sweetened diet group. a = *P* < 0.05 vs. NC and b = *P* < 0.05 vs. corresponding energy groups. Values represent the means ± SEM, n = 3–6

Figure 7 shows result of the effect of sugar and natural honey sweetened diets on serum TNF-α concentration in female and male rats. The data obtained showed a dose-dependent increase of TNF-α (ng/L) in the female rats fed sugar-sweetened diets, S8% and S16% by 29.9 and 47.7% respectively relative to the normal control and by 54.6 and 55.7% respectively, compared to the rats fed honey-sweetened diets, H10% and H20% (*P* < 0.05). Compared to the control, TNF-α was decreased in the female rats fed honey-sweetened diets (*P* < 0.05). As in the female rats, TNF-α in the male rats fed sugar-sweetened diets S8% and S16% was found to increase dose-dependently by 32.8 and 47.1% respectively compared to the normal control and by 50.1 and 56.3% respectively compared to the corresponding honey-sweetened diets fed groups (*P* < 0.05). Compared to the control, TNF-α of the honey sweetened diets i.e. H10% and H20% was decreased by 25.7 and 17.4%.

## Discussion

This study was designed to evaluate and compare the effect of 29-week feeding of natural honey and table sugar sweetened diets on energy metabolism/homeostasis of male and female Wistar rats. Daily dietary consumption was measured in order to provide a platform for comparison of the sweeteners. There were no significant differences in the quantities of sugar sweetened diet consumed compared to the honey in both male and female sub-groups, except the H20% diet fed female rats, where consumption decreased over time. Free fructose, a major component of honey has been reported to lower food intake [18] owing to its delayed gastric emptying property [19]. The increased fructose at higher concentration may be responsible for the observed reduced consumption. However, in moderate amount both sweeteners did not affect diet intake significantly. This observation is in line with report of Chepulis and colleagues [20] who showed that at moderate amounts, sugar and honey (7.9% sucrose and 10% honey) did not significantly affect diet intake.

In the female rats, and at higher percent sweetener incorporation, both honey (H20%) and sugar (S16%) sweetened diets caused significant successive increases in body weight gain compared with the control, suggesting a direct relationship between body weight gain and amount of sweetener in the diet. However, the increases caused by the H20% diet was relatively greater than the S16%, even though lesser amount of the former were consumed compared to the latter. A close observation of the result reveals that the female rats fed the sugar-based diet initially lost body weight in the first 2–3 weeks, due probably to dietary adaptation and adjustment which was not the case with the H20%. Adjusting for this initial weight lost in the S16% fed animals would place its effect at par with the of H20% on body weight gain. The relative white adipose tissue (WAT) data buttresses the body weight result - no significant difference in the wet weight of the adipose among the treatment groups. In the male rat groups, 8% sugar-based diet caused significant body weight increase compared to the control and 10% honey-based diet. This is consistent with an earlier report that in moderate incorporation honey-based diet (10%) significantly reduced body weight compared to a sucrose-based diet (7.9%) when fed for 52 weeks [20]. More so, it was also demonstrated lower weight gain of rats fed diets containing honey compared to sucrose [21]. Although the exact mechanism by which honey decreases weight gain is not fully understood, data from some studies indicate that honey might reduce weight gain by modulation of appetite-regulating hormones such as leptin, ghrelin and peptide YY [22].

Fasting blood glucose was measured over the study period to assess the effect of the sweetened diets on blood glucose regulation. Aside occasional transient differences, there were no significant changes in the study groups; males and females compared to the control, suggesting a no disparity of effect of sugar and honey-based diets on blood glucose within the study period. In part, the fasting blood glucose data in this study agrees with the findings of [10] where 13-week dietary supplementation with natural honey was found not to cause any significant change in FBG. Hence based of blood glucose data alone, the preference of sweetening the diet with honey over table sugar appears unfounded. Moreover, our current observation is at variance with some earlier reports that honey exerts hypoglycaemic action in healthy and diabetic individuals [23, 24]. It is pertinent to note that almost all the studies reporting hypoglycaemic effect of honey was administered in short term, the maximum being 12 weeks. This perspective is essential considering the fact that diabetes is a chronic disorder.

The measured wet weights of internal organs of the female rats showed no significant effect of the study diets on the relative weights of heart, liver, kidneys compared with the control. In the male rats, there was no significant difference in relative heart, liver and brain weight in test groups compared to normal control. The sugar-fed group (S16%) relative liver weight was significantly higher than normal control and the corresponding honey-fed group (H20%). This may suggest liver hypertrophy or inflammatory response to high levels of the sweeteners in the diet [25]. However, further studies involving liver histology and liver function test is needed for a conclusive report.

In the current study, the leptin concentrations of rats fed the sugar-based diet increased significantly in a dose-dependent manner when compared to the corresponding honey-fed groups and the normal control in female rats. This finding is consistent with the finding of Carmody and colleagues [20] who reported reduced serum leptin in honey fed Sprague Dawley rats after 33 days. The increased leptin concentration may be as a result of desensitization of leptin signals via increased insulin level in sugar-fed group as seen in this study. Leptin levels in the male test rats were not different from the control. Sex disparity in leptin has been well documented [26–28] which usually develop at puberty and is believed to be triggered by sex hormones. Other factors include larger adipose tissue mass and leptin production rate per unit mass of adipose tissue females [29].

This study observed an elevated TNF-α level in the rats fed sugar-sweetened diet, unlike the reduced TNF-α level in the honey-fed rats of both sexes when compared to the control. This might suggest a probable tendency of sugar-sweetened diet to cause insulin resistance. TNF-α, which was initially considered a factor involved in necrosis of tumour cells has now been implicated in the cause of insulin resistance and obesity [30, 31]. In obese rodents and humans, the expression of TNF-α is reportedly increased and is correlated with insulin resistance and adiposity [32].

Insulin level was significantly high in animals fed sugar-sweetened diets compared to the corresponding honey-fed groups and normal control in both the male and female rats. This finding is at variance with earlier reports by Carmody et al. [20] who reported no significant difference in insulin levels of honey-fed rats compared to sucrose fed rats after a 33-day feeding. This difference may be due to botanical and geographical sources of honey and the length of study. The increase in insulin may also be as a result of insulin resistance which is also supported by the observed significant increase in TNF-α (TNF-α induces insulin resistance by inhibiting insulin signal transduction) and leptin levels (females only) in sugar fed rats compared to honey fed ones.

## Conclusion

In sum, the results of this study tend to associate long term sugar consumption than the Obudu natural honey with obesity and inflammatory pathologic processes in rats. It however, did not show a non-obesigenic effect with the honey, but sublime effects that only require more time to precipitate obesity. The implications are largely similar in male and females, but with a tilt more towards the female, given the leptin data. However, further studies are required to confirm this.

## Supplementary information

**Additional file 1.** Raw data obtained in the study. This additional file contains all data generated during this study and their transposition into charts/figures. They include data for body weights, tissue/organ weights, diet consumed, blood glucose, serum leptin and tissue necrosis factor-alpha (TNF-α) as measured in both male and female animal groups. NB: There is no provision for such a file to be cited in the main text, rather, it is for reviewers and editors’ perusal.

## Data Availability

All data generated or analysed during this study are included in this published article [and its supplementary information files (Additional file 1]).

## Abbreviations

ELISA: Enzyme linked immunosorbent assay
FAREC-FBMS: Faculty Animal Research Ethics Committee, Faculty of Basic Medical Sciences
FBG: Fasting blood glucose
H10%: Ten percent (10%) honey diet
H20%: Twenty percent (20%) honey diet
HRP: Horseradish peroxidase
NC: Normal control
S16%: Sixteen percent (16%) sugar diet
S8%: Eight percent (8%) sugar diet
TNF-α: Tissue necrosis factor - alpha
WAT: White adipose tissues
